# Amiodarone-Induced Thyrotoxicosis Presenting as Refractory Atrial Fibrillation in Hypertrophic Cardiomyopathy: A Case Report

**DOI:** 10.7759/cureus.101415

**Published:** 2026-01-13

**Authors:** Sofia Sequeira, Susana Correia, Adriana Santos, Maria Beatriz Santos

**Affiliations:** 1 Internal Medicine, Hospital de Santo Espírito da Ilha Terceira, Angra do Heroísmo, PRT

**Keywords:** amiodarone-induced thyrotoxicosis, antiarrhythmic therapy, atrial fibrillation, heart failure with preserved ejection fraction, hypertrophic cardiomyopathy

## Abstract

Amiodarone-induced thyrotoxicosis (AIT) is an uncommon but potentially severe adverse effect that may significantly compromise arrhythmic control. Thyroid dysfunction induced by amiodarone can precipitate or perpetuate atrial fibrillation, particularly in patients with underlying structural heart disease. We report the case of a 54-year-old woman with non-obstructive hypertrophic cardiomyopathy, heart failure with preserved ejection fraction, and paroxysmal atrial fibrillation, who developed recurrent episodes of atrial fibrillation with rapid ventricular response refractory to conventional rate and rhythm control strategies. Despite repeated exposure to amiodarone, thyroid function tests revealed severe thyrotoxicosis with suppressed thyroid-stimulating hormone and markedly elevated free thyroid hormone levels. Endocrinological evaluation supported the diagnosis of type 2 or mixed AIT, and combined therapy with antithyroid drugs and systemic corticosteroids was initiated. The patient continued to experience recurrent arrhythmic episodes despite withdrawal of amiodarone and aggressive pharmacological management, highlighting the complex bidirectional relationship between thyroid dysfunction and cardiac arrhythmias. This case underscores the importance of early recognition of AIT in patients with difficult-to-control atrial fibrillation and emphasizes the need for a multidisciplinary approach involving cardiology and endocrinology.

## Introduction

Amiodarone is a widely prescribed class III antiarrhythmic agent with proven efficacy in the management of both supraventricular and ventricular arrhythmias, particularly atrial fibrillation in patients with structural heart disease. Despite its clinical effectiveness, amiodarone is associated with a broad spectrum of extracardiac adverse effects, most notably thyroid dysfunction, due to its high iodine content and direct cytotoxic effects on thyroid follicular cells [1-3].

Amiodarone-induced thyrotoxicosis (AIT) is a potentially life-threatening complication and is associated with increased cardiovascular morbidity and mortality, especially in patients with underlying heart disease [2,4]. Two main subtypes of AIT have been described. Type 1 AIT results from iodine-induced excessive thyroid hormone synthesis, typically occurring in patients with latent or overt thyroid disease, whereas type 2 AIT represents a destructive thyroiditis in an otherwise normal thyroid gland, leading to the release of preformed thyroid hormones [2,5]. Mixed or indeterminate forms are increasingly recognized and often complicate both diagnosis and management [5,6].

Thyroid hormone excess exerts profound cardiovascular effects, including increased heart rate, enhanced myocardial contractility, shortened atrial refractory periods, and heightened sensitivity to catecholamines, all of which contribute to an increased risk of atrial fibrillation. These effects are mediated by upregulation of β-adrenergic receptors, increased expression of cardiac ion channels, and shortening of atrial action potential duration, leading to enhanced atrial automaticity and electrical instability [7,8]. Even subclinical hyperthyroidism has been associated with a significantly higher incidence of atrial fibrillation and adverse cardiovascular outcomes [9].

In patients with hypertrophic cardiomyopathy, who are particularly dependent on atrial contraction for adequate ventricular filling, these effects may precipitate severe hemodynamic compromise and render atrial fibrillation refractory to standard therapeutic strategies [7,10].

Although amiodarone-induced thyroid dysfunction is a well-recognized complication, its timely diagnosis may be significantly influenced by healthcare access and socioeconomic factors. Regular monitoring of thyroid function is recommended for patients receiving long-term amiodarone therapy; however, adherence to surveillance protocols may be suboptimal in resource-limited or low-income settings, where access to laboratory testing and specialized follow-up is often restricted [2-4]. Emerging evidence suggests that disparities in healthcare access may contribute to delayed recognition of amiodarone toxicity and an increased burden of related cardiovascular complications, including worsening atrial fibrillation and heart failure decompensation [3,4,9]. These considerations underscore the importance of structured follow-up and equitable access to monitoring for patients treated with amiodarone.

We report a case of refractory atrial fibrillation secondary to AIT in a patient with non-obstructive hypertrophic cardiomyopathy, highlighting the diagnostic challenges and complex therapeutic decisions inherent to this clinical scenario.

## Case presentation

A 54-year-old woman, fully independent in daily activities, with a known history of non-obstructive hypertrophic cardiomyopathy and paroxysmal atrial fibrillation, was referred to our cardiology department for further management of recurrent symptomatic arrhythmia. Previous cardiac imaging had demonstrated marked left atrial dilatation, a non-dilated left ventricle with preserved global and segmental systolic function, concentric left ventricular hypertrophy predominantly involving the interventricular septum, absence of a significant intraventricular gradient, and grade III diastolic dysfunction with a restrictive filling pattern. She had heart failure with preserved ejection fraction. Prior coronary angiography excluded obstructive coronary artery disease. Additional comorbidities included type 2 diabetes mellitus and obesity.

**Figure 1:**
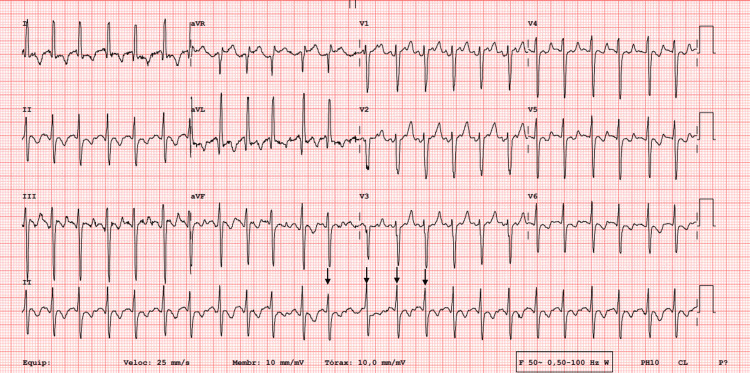
Twelve-lead electrocardiogram obtained during hospitalization demonstrating atrial fibrillation with rapid ventricular response. Irregularly irregular RR intervals, and a ventricular rate exceeding 140 beats per minute. AF: atrial fibrillation; RR: interval between successive R waves

Before referral, the patient experienced multiple symptomatic episodes of atrial fibrillation with rapid ventricular response, presenting with palpitations, chest discomfort, dyspnea, fatigue, tremors, and presyncope. During hospitalization, electrocardiography documented atrial fibrillation with rapid ventricular response, with ventricular rates exceeding 140 beats per minute (Figure 1). Previous treatment with beta-blockers and amiodarone had achieved only transient rate and rhythm control. Due to recurrent symptomatic arrhythmia, she was referred to a tertiary center for catheter ablation.

During the pre-procedural evaluation at the referring institution, thyroid function tests revealed severe thyrotoxicosis. AIT was suspected, amiodarone was discontinued, and treatment with thiamazole and systemic corticosteroids was initiated before our evaluation.

At the time of assessment in our department, follow-up laboratory testing showed persistently suppressed thyroid-stimulating hormone levels and elevated free thyroid hormone concentrations, despite partial biochemical improvement under therapy (Table 1).

**Table 1:** Thyroid function tests following initiation of antithyroid therapy. TSH: thyroid-stimulating hormone; Free T4: free thyroxine; Free T3: free triiodothyronine

Time point	TSH (µIU/L)	Free T4 (pmol/L)	Free T3 (nmol/L)
At referral	0.006	99	11.7
Follow-up	0.014	26	1.3

Despite ongoing antithyroid and corticosteroid therapy, the patient continued to experience recurrent episodes of atrial fibrillation with rapid ventricular response. Multiple attempts at rate and rhythm control using high-dose beta-blockers, propafenone, intravenous beta-blockers, and digoxin were unsuccessful, with persistent ventricular rates frequently exceeding 140 beats per minute. Endocrinological reassessment supported the diagnosis of type 2 or mixed AIT.

## Discussion

AIT represents a clinically significant and often underrecognized cause of secondary thyrotoxicosis, particularly in patients receiving long-term amiodarone therapy for atrial fibrillation [2,3]. This case illustrates a well-known but challenging therapeutic paradox in contemporary cardiology: amiodarone, one of the most effective antiarrhythmic drugs for atrial fibrillation, may itself precipitate thyroid dysfunction that exacerbates arrhythmic instability and undermines rhythm and rate control [1,4].

Excess thyroid hormone promotes atrial fibrillation through multiple mechanisms, including upregulation of β-adrenergic receptors, shortening of atrial action potential duration, increased atrial automaticity, and enhanced atrial ectopic activity [7,8]. In addition, thyrotoxicosis induces a hyperdynamic circulatory state characterized by tachycardia, increased myocardial oxygen consumption, and reduced diastolic filling time [9]. These effects are particularly deleterious in patients with hypertrophic cardiomyopathy and advanced diastolic dysfunction, in whom ventricular filling is highly dependent on atrial systole. Consequently, the combination of loss of atrial contraction and rapid ventricular response may result in marked clinical deterioration despite preserved systolic function.

Importantly, the arrhythmogenic effects of thyroid hormone excess are not confined to overt thyrotoxicosis. Large population-based studies have demonstrated that even subclinical hyperthyroidism is associated with a significantly increased risk of atrial fibrillation and cardiovascular events, underscoring the clinical relevance of modest elevations in thyroid hormone levels [10]. In the present case, severe biochemical thyrotoxicosis likely acted as a dominant driver of arrhythmic refractoriness, overriding conventional pharmacological rate and rhythm control strategies.

Differentiation between type 1 and type 2 AIT is crucial but often challenging in routine clinical practice. Type 1 AIT is characterized by iodine-induced hormone overproduction, whereas type 2 AIT results from a destructive inflammatory process within the thyroid gland [2,5]. Mixed or indeterminate forms are increasingly recognized, particularly in patients with prolonged or intermittent exposure to amiodarone, and frequently necessitate combined therapeutic approaches [5,6]. In this patient, the absence of known pre-existing thyroid disease, negative antithyroid antibodies, severe biochemical thyrotoxicosis, and partial response to corticosteroid therapy favored a diagnosis of type 2 or mixed AIT. Mild positivity of thyroid-stimulating hormone receptor antibodies introduced diagnostic uncertainty but was insufficient, in isolation, to support Graves’ disease.

Management of atrial fibrillation in the setting of active thyrotoxicosis remains particularly challenging. Beta-blockers are recommended as first-line therapy for symptom control and ventricular rate reduction; however, they may be insufficient in severe thyrotoxicosis due to persistent adrenergic stimulation and advanced atrial remodeling [7,9]. Reintroduction of amiodarone may appear clinically attractive in refractory cases but risks perpetuating thyroid hormone excess and sustaining a vicious cycle of endocrine and electrical instability [1,3].

Corticosteroids represent the cornerstone of treatment for type 2 AIT, aiming to reduce thyroid inflammation and hormone release, while thionamides are indicated in type 1 or mixed forms [2,5,11]. Nevertheless, clinical and biochemical improvement often requires several weeks, and relapse during steroid tapering is well documented [11]. This temporal dissociation between biochemical recovery and arrhythmic stabilization underscores the importance of early recognition of AIT and close collaboration between cardiology and endocrinology teams to optimize outcomes and prevent recurrent hospitalizations.

## Conclusions

AIT should be actively considered in patients with atrial fibrillation that becomes refractory to standard rate or rhythm control, particularly in those with underlying structural heart disease. This case highlights how thyroid hormone excess can precipitate and perpetuate atrial fibrillation, creating complex diagnostic and therapeutic dilemmas. What makes this case distinctive is the occurrence of severe, refractory atrial fibrillation in the setting of type 2 or mixed AIT in a patient with non-obstructive hypertrophic cardiomyopathy, a population highly dependent on atrial contraction for hemodynamic stability. Despite prompt recognition, withdrawal of amiodarone, and combined endocrine therapy, arrhythmic control remained challenging, underscoring the profound and sustained arrhythmogenic impact of thyroid hormone excess. Early recognition of AIT, prompt discontinuation of amiodarone, and coordinated multidisciplinary management are essential to interrupt the cycle of endocrine dysfunction and arrhythmic instability and to improve clinical outcomes.
